# Polarity-specific modulation of pain processing by transcranial direct current stimulation – a blinded longitudinal fMRI study

**DOI:** 10.1186/s10194-018-0924-5

**Published:** 2018-10-24

**Authors:** Steffen Naegel, Josephine Biermann, Nina Theysohn, Christoph Kleinschnitz, Hans-Christoph Diener, Zaza Katsarava, Mark Obermann, Dagny Holle

**Affiliations:** 1 Department of Neurology, University of Duisburg-Essen, University Hospital Essen, Hufelandstr. 55, 45122 Essen, Germany; 2 Institute of Diagnostic and Interventional Radiology and Neuroradiology, University of Duisburg-Essen, University Hospital Essen, Hufelandstr. 55, 45122 Essen, Germany; 3 Department of Neurology, Evangelical Hospital Unna, Holbeinstr. 10, 59423 Unna, Germany; 4 Center for Neurology, Asklepios Hospitals Schildautal, Karl-Herold-Straße 1, 38723 Seesen, Germany; 5 EVEX Medical Corporation, 40 Vazha-Pshavela Avenue, Tbilisi, 0177 Georgia; 6 Sechenov University Moscow, 8-2 Trubetskaya str., Moscow, 119991 Russian Federation

**Keywords:** Neuromodulation, Nociception, Pain, fMRI, tDCS, Transcranial direct current stimulation

## Abstract

**Background:**

To enrich the hitherto insufficient understanding regarding the mechanisms of action of transcranial direct current stimulation (tDCS) in pain disorders, we investigated its modulating effects on cerebral pain processing using functional magnetic resonance imaging (fMRI).

**Methods:**

Thirteen right-handed healthy participants received 20 min of 1.5 mA tDCS applied over the primary motor cortex thrice and under three different stimulation pattern (1.anodal-tDCS, 2.cathodal-tDCS, and 3.sham-tDCS) in a blinded cross-over design. After tDCS neural response to electric trigeminal-nociceptive stimulation was investigated using a block designed fMRI.

**Results:**

Pain stimulation showed a distinct activation pattern within well-established brain regions associated with pain processing. Following anodal tDCS increased activation was detected in the thalamus, basal ganglia, amygdala, cingulate, precentral, postcentral, and dorsolateral prefrontal cortex, while cathodal t-DCS showed decreased response in these areas (p_FWE_ < 0.05). Interestingly the observed effect was reversed in both control conditions (visual- and motor-stimulation). Behavioral data remained unchanged irrespective of the tDCS stimulation mode.

**Conclusions:**

This study demonstrates polarity-specific modulation of cerebral pain processing, in reconfirmation of previous electrophysiological data. Anodal tDCS leads to an activation of the central pain-network while cathodal tDCS does not. Results contribute to a network-based understanding of tDCS’s impact on cerebral pain-processing.

## Background

Experimental and non-experimental pain causes activation in a complex neuronal network previously reported as pain neuromatrix, reflecting the multidimensionality of pain [1]. Sensory-discriminative components of pain are processed by primary (S1) and secondary (S2) somatosensory cortices, the thalamus, and posterior part of the insula in the lateral pain system [2], while affective-motivational components are processed in the medial pain-system including the anterior cingulate cortex (ACC), and anterior parts of the insula [3–6]. Several other brain areas are involved in motor, cognitive and autonomic aspects of pain, as well as pain modulation [2, 3, 7–10].

Some pain and headache disorders appear to be caused by a dysbalanced network [11], and neuromodulatory approaches are increasingly used therapies. Transcranial direct current stimulation (tDCS) is a non-invasive, safe, and painless technique which is applied in various chronic pain disorders such as fibromyalgia [12], spinal cord injury pain [13] and menstrual migraine [14]. TDCS modulates activity in brain regions specific to the site of application and stimulation parameters. For anodal stimulation a raised and for cathodal stimulation a decreased level of cortical excitability at the targeted brain area was previously shown [4, 15]. Pain reduction caused by stimulation of the primary motor-cortex may result from modulation of the pain processing network, but so far is only insufficiently understood.

## Methods

The aim of this study was to assess the effect of tDCS on cerebral pain processing using functional magnetic resonance imaging (fMRI) and to identify the brain areas involved in this neuro-modulation.

Thirteen (6 women) healthy subjects were investigated using functional resonance magnetic imaging (fMRI). Inclusion criteria were age over 18 years and right-handedness. Exclusion criteria were primary headache-syndromes and other pain conditions as well as psychiatric or other somatic illnesses. All thirteen participants did not experience any pain or injuries during the study period and four weeks prior to study inclusion, and all were advised to prevent sleep-deprivation before study participation and to not take any alcohol, central acting drugs or pain medication for at least 24 h before each experiment.

All participants gave their written informed consent according to the Declaration of Helsinki prior to study inclusion. The local ethics committee of the University of Duisburg-Essen approved the study protocol.

Due to the small number of fMRI studies and tDCS no formal power calculation was performed. The sample size was estimated corresponding to previous tDCS studies using fMRI.

### Study design and fMRI-data acquisition

**Fig. 1:**
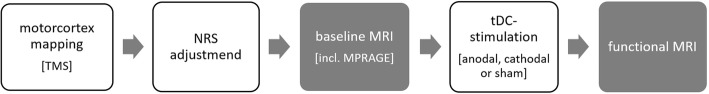
Study sequence per appointment. Illustration of the study sequence for each appointment (3 per subject), white boxes represent activity outside the scanner including motorcortex mapping using transcranial magnetic stimulation, pre DCS stimulus intensity adjustment (NRS = 5), and DC Stimulation (anodal, cathodal or sham in pseudorandomized order). Grey boxes represent MRI measurements including anatomical and functional MRI)

Imaging was performed using a 3 Tesla MRI scanner (Magnetom Skyra, Siemens Healthcare, Erlangen, Germany) equipped with a standard 20-channel head/neck coil. All participants underwent the standardized scanning procedure three times. The order of the DC-Stimulation (sham, anodal, and cathodal) was pseudorandomly preassigned for each subject and subjects were blinded regarding stimulation type at any time. Between the scanning time points a two-month interval was kept to prevent carryover-effects. Imaging included T1 (magnetization prepared rapid acquisition gradient echo, MPRAGE), and functional magnetic resonance imaging (EPI, 3x3x3mm, 52 Slices, FOV-read = 240 mm, TR =3020 ms TE = 26 ms flip-angle 90°). Order of sequences including DC-stimulation was equal on every appointment and is illustrated in Fig. 1.

Prior to analysis all images were rated regarding image quality and pathologies. This was double-checked by an experienced neuro-radiologist (N.T.) and found to be unremarkable in all subjects.

FMRI was performed using a block-design with 7 images per task/stimulus-epoch (=21,14 s), and baseline periods with a duration 13 images (=39,26 s). Three different conditions (A, B, C) were investigated.

- A: Nociceptive stimuli (11 blocks/epochs per session) were applied to the right forehead by a special copper platinum planar concentric electrode (Walter Graphtek, Luebeck, Germany, http://www.walter-graphtek.com/) 10 mm above the entry zone of the supraorbital nerve. It was previously shown that this setup is able to specifically depolarize C-Fibers and thereby, is pain specific [16]. In each epoch 7 nociceptive stimuli (=one per image) were administered. Each stimulus was applied as pulse-train of five pules (temporal summation, monopolar square wave, duration 0.5 ms, pulse interval 5 ms, pulse length: 1000 μs, V_max_: 400 V). Pulses were generated by a commercially available high voltage constant current stimulator (DS7AH**,** Digitimer Ltd., Welwyn Garden City, England, UK). Stimulus intensity was adjusted to a numeric rating scale (NRS) of 5/10 by setting the amperage before MRI and tDCS procedure. During fMRI subjects noted the experienced pain intensity after every stimulation block applying NRS (0–10) within a 6 s rating period.
- B: Visual stimulation (2 blocks/epochs per session) using a build in projection-system rearwards the MRI was applied with a 20 × 15 square checkerboard matrix alternation (frequency of 4 Hz over 7 consecutive images) projected with a size of 100cmx75cm covering the complete field of view out of the 70 cm MR-Bore.
- C: The third condition was a motor task (3 blocks per session). Subjects were instructed to tap the left index and thumb a frequency of 1 Hz. Visual instruction was given for 7 consecutive images and subjects were advised to quickly re-close eyes during these blocks and to stop tapping when scanner room darkens again. Accuracy was controlled from scanners anteroom.

### Functional MRI data processing and analysis

Functional MRI data processing and analysis was performed using SPM8 (Wellcome Trust Centre for Neuroimaging, UCL, London, UK [https://www.fil.ion.ucl.ac.uk/spm/]) and MATLAB (Matlab R2015a, The MathWorks, Natick, MA, USA). First four scans were excluded to prevent tampering due to general scanner drift. Pre-processing included “realign and unwarp”, co-registration of the structural and mean functional image, normalization into the Montreal Neurological Institute (MNI)-space by segmentation of the anatomical image, and normalization of the co-registered EPI images. Spatial smoothing was performed with an isotropic Gaussian kernel of 8 mm full-width at half maximum [17].

First level statistics was performed using a general linear model with repeated box-cars convolved with a hemodynamic response function provided with SPM8. In that model all three conditions as well as the 6 s rating period were considered. To compensate for the presence of movement artefacts, movement parameters were included as covariates.

To assess activation pattern of the conditions (trigeminal nociception, visual stimulation, and motor activity) a primary, explorative random-effects group analysis was performed averaging all DC-stimulation types with a whole brain family wise error corrected (FWE) threshold of *p* < 0.05.

To investigate effects of the tDC-stimulation on cerebral activation a definite second level statistic was performed using a longitudinal random-effects model feed with the results from first level for trigeminal nociception, visual, and motor-tasks. To investigate the maximum effect on BOLD (blood oxygen level depended)-signal changes induced by the applied tDC-stimulation, a direct comparison of anodal and cathodal stimulation was calculated. For the evaluation of trigeminal nociception, we a priori identified regions generally accepted to be involved in pain processing as described previously [18]. Anatomical regions of interest (ROIs) were derived from “automated anatomic labeling toolbox”-templates (AAL) for the thalamus, amygdala, basal ganglia (combined AAL template: caudate + pallidum + putamen), dorsolateral prefrontal, insular, supplementary motor, primary somatosensory, cingulate cortex (combined AAL template: cingulum_ant + cingulum_mid), and the cerebellum (combined AAL template: Cerebelum_Crus1 + 2, Cerebelum3–10, and Vermis_1–10) using marsbar [19, 20]. As the left primary motorcortex was directly targeted by tDCS this (combined AAL template: paracentral_Lobule_L + precentral_L) was additionally added as ROI. Significance level for exploratory analysis was set to p_unc_ < .0005. Region of interest analysis was applied using Family-Wise-Error correction with a significance level of p_FWE_ < .05.

For all fMRI data, only results surviving corrected thresholding with p_FWE_ < 0.05 are reported and discussed (Exception: Table 2 additionally presenting results from the exploratory analysis, p_unc_ < .0005).

### Statistical analysis on clinical and demographic data

For demographics and behavioral data ANOVA (*p* < 0.05) with post-hoc Bonferroni analysis was performed with IBM SPSS Statistics Version 22 (International Business Machines Corporation, Armonk, New York, USA).

### DC-stimulation

After pre-imaging (e.g. MPRAGE) subjects left the scanner and received DC-Stimulation of the left primary motor cortex (M1) applied by HDCstim (Newronika s.r.l., Milan, Italy) equipped with two saline-soaked sponge covered electrodes (anode: 5x5cm^2^; cathode: 6 × 8.4cm^2^). Stimulation was performed for 20 min with an intensity of 1,5 mA and a current ramp of 7 s. The opposite electrode was placed over the contralateral supraorbital region. The primary motor-cortex was mapped using transcranial magnetic stimulation (TMS) (MagPro X100, MagVenture Inc., Atlanta, GA, USA) identifying the representation of the right first dorsal interosseous muscle (IOD1) one hour before MR-recording. TMS was performed using a figure-of-eight coil (handle directed rearwards) and superficial MEP recording. Three tDCS application modes were used: 1.) anodal (a-tDCS), 2.) cathodal (c-tDCS) and 3.) sham (s-tDCS). For s-tDCS 30 s of anodal stimulation was delivered and afterwards ceased without participants knowledge, which is an established blinding method [21, 22]. The M1 was targeted as in the current literature it is the most convincing stimulation-region regarding clinical and experimental pain [23–25].

## Results

### Demographic and behavioral data

We investigated 13 healthy subjects (6 female). All subjects were right-handed and average age was 23,92 (± 1.98 SD) years. No subject suffered from any relevant illnesses, including pain and headache disorders. Analysis of collected behavioral data did not show any significant differences in pain ratings on a verbal rating scale of zero to ten (0 = no pain, 10 = worst imaginable pain; *p* = 0.377, NRS anodal 6.81 ± 1.58, cathodal 5.99 ± 1.53, sham 6.55 ± 1.43) nor in applied nociceptive stimulus intensity (*p* = 0.995, anodal 1.31 ± 0.97 mA, cathodal 1.28 ± 0.98 mA, sham 1.28 ± 0.98 mA).

### Functional magnetic resonance imaging (fMRI)

#### Explorative data assessment

Single subject activations (not provided) and explorative primary group analyses showed cerebral activation pattern consistent with anatomical knowledge and previously reported results for pain-, motor- and visual-processing (Table 1).

**Table 1:** Average fMRI activation

MNI X Y Z	Anatomical area		k_E_	T
**A – Nociceptive processing**				
-30–58 -26	L	Cerebellar hemisphere	6763	10.22
26–58 -24	R	Cerebellar hemisphere		9.47
6–64 -16	BL	Cerebellar vermis		9.47
-38–12 56	L	Motor cortex	11,434*	9.09
-2 2 56		Suppl. Motor area /SMA		9.02
-62–20 18		Somatosensory cortex (head)		8.94
64–16 20	R	Somatosensory cortex (head)	7887†	8.82
60 12 0		Rolandic operculum		8.80
58 12 18		Frontal inf. Operculum		7.03
42 46 18	R	Dorsolateral-prefrontal-cortex	331	5.67
36 50 24				5.24
-34 44 28	L	Dorsolateral-prefrontal-cortex	45	5.08
-10–22 48	L	Cingulum	48	5.07
40–14 -6	R	Posterior insula	35	4.95
46–26–4		Superior temporal gyrus		4.90
52–38 -16	R	Middle temporal gyrus	46	4.89
56–48 -18		Inferior temporal gyrus		4.83
**B – Visual processing**				
-8–88 0	BL	Calcarine gyrus	32,000‡	18.86
4–88 -2		Lingual gyrus		17.93
6–80 -2				17.31
0–54 -36	BL	Cerebellar vermis	91	7.34
50–4 54	R	Middle Frontal Gyrus	53	5.30
**C – Motor processing**				
38–18 58	R	M1/Precentral Gyrus	2438	20.67
50–16 52				12.48
-16–50 -20	L	Cerebellar hemisphere	1502	15.62
-58–18 48	L	Pre- + Postcentral Gyrus	113	6.61
8–4 54	R	Suppl. motor cortex	226	6.52
6 0 66				5.39
-8–88 0	BL	Calcarine gyrus	800	6.47
8–98 -4				5.82
10–82 4				5.23
16–20 6	R	Thalamus	42	5.77

Averaged BOLD responses (respecting all three tDC-stimulation paradigms to a third) to A nociceptive stimulation, B visual stimulation and C motor activation (incl. visual instruction); All results are whole brain Family-Wise-Error corrected (p_FWE_ < 0.05). Fused blobs: *including activation in the thalamus, anterior and posterior insular cortex, † including activation in the thalamus, anterior insular cortex and basal ganglia. ‡ widespread bilateral activation of V1, visual thalami and downstream visual cortices. R = right, L = left, BL = bilateral, k_E_ = cluster extend; MNI = Montreal Neurological Institute

#### BOLD-modulation by DC-stimulation

Comparison of the different stimulation modalities revealed stimulation mode dependent activation/BOLD-response differences for trigeminal nociception, motor and visual stimulation.

### Modulation of nociceptive processing

**Fig. 2:**
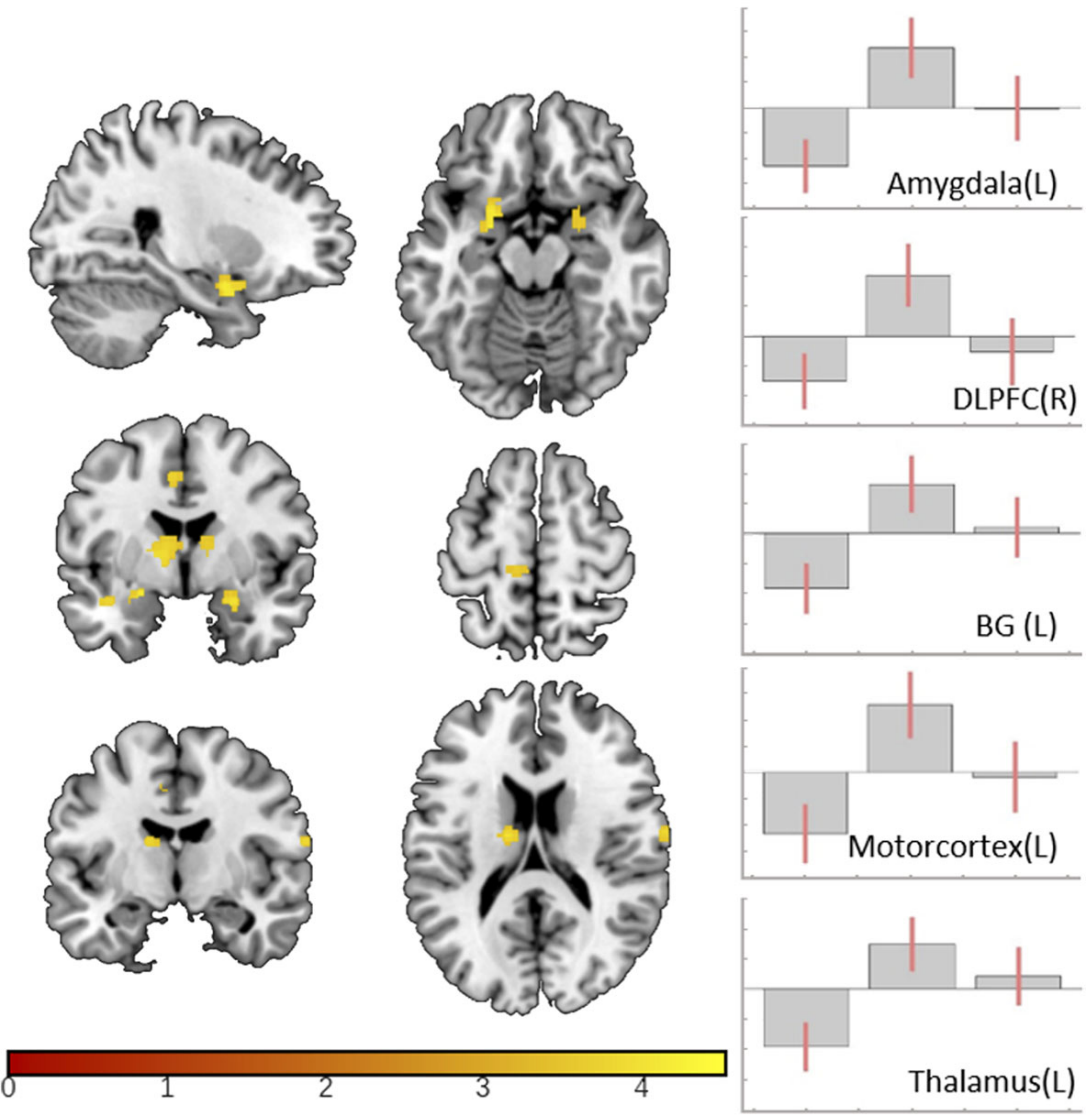
Polarity dependent effect of tDCS on trigeminal nociceptive processing. Visualization of tDC-stimulation induced BOLD-response alterations in trigeminal nociceptive processing (post-anodal vs. post-cathodal) superimposed on MRICONs ch2bet-template, thresholded at p_unc_ < 0.0005. Corresponding contrast estimates in the following order: 1. postcathodal, 2. postanodal and 3. postsham. Anatomical areas from top to bottom: bilateral amygdala, left cingulate cortex, bilateral basal ganglia, left motorcortex, left temporal lobe, left thalamus and right postcentral gyrus

Comparing anodal with cathodal stimulation a BOLD response increase for anodal stimulation was detected in multiple pain processing areas including bilateral amygdala and basal ganglia, left sided thalamus, cingulate cortex, premotor and motor cortex and right sided dorsolateral prefrontal and postcentral cortex (Table 2; Fig. 2). No decrease of BOLD-response was seen in this comparison (post-anodal < post-cathodal). Investigating the contrast estimates of the regions represented by peak voxels it becomes obvious that cathodal DC-stimulation leads to a decreased and anodal DCS to an increased BOLD response in the investigated pain processing areas, while BOLD-signal-intensities after sham-stimulation were found to be in between of those two conditions (Fig. 2). When directly contrasting active (anodal and cathodal) to sham stimulation the modulation of the BOLD response did not reach the defined significance threshold.

**Table 2:** Effect of tDCS on trigeminal nociceptive processing

MNI X Y Z	Anatomical area		k_E_	T	p_FWE_ cluster	p_FWE_ peak
−22 8–16	L	Amygdala^a^	107	4.48	.005	.001
−12 2 4	L	Basal ganglia^a^	208	4.24	.033	.006
26 0–20	R	Amygdala^a^	125	4.18	.002	.001
56–30 -8	R	Mid. temporal gyrus	74	4.17	NA	NA
−10 -28 70	L	M1 and premotor cortex^a^	162	4.11	.010	.012
−14 -10 18	L	Thalamus^a^	39	3.99	.015	.008
56–18 6	R	Superior temporal gyrus	86	3.97	NA	NA
14 4 6	R	Basal ganglia^a^	94	3.93	.027	.029
−50 -32 12	L	Superior temporal gyrus	49	3.85	NA	NA
66–8 18	R	Postcentral gyrus^a^	24	3.81	.042	.025
−40 -2 -18	L	Posterior Insular cortex	27	3.77	NS	NS
−6 -4 44	L	Cingulate cortex^a^	47	3.77	.03	.027
10–24 74	R	Suppl. motor/premotor cortex	25	3.73	NS	NS
4 14 0	R	Basal ganglia / Caudate ncl.^a^	59	3.68	.027	.032
38 2 54	R	DLPFC^a^	44	3.67	.044	.047
44 42 4	R	DLPFC	42	3.58	NS	NS

Areas with significant DC-stimulation induced alterations (postcathodal vs. postanodal). Illustration in Fig. 2. Exploratory significance level p_unc_ < .0005. Additional region of interest analysis (ROI) with applied Family-Wise-Error correction for the neuropain-matrix as indicated in the materials and method section (^a^p_FWE_ < 0.05)

R = right, L = left, BL = bilateral, Ke = cluster extend, MNI = Montreal Neurological Institute: NA = not applicable, NS = not significant. M1 = primary motorcortex, DLPFC = dorsolateral prefrontal cortex

### Modulation of control conditions

**Fig. 3:**
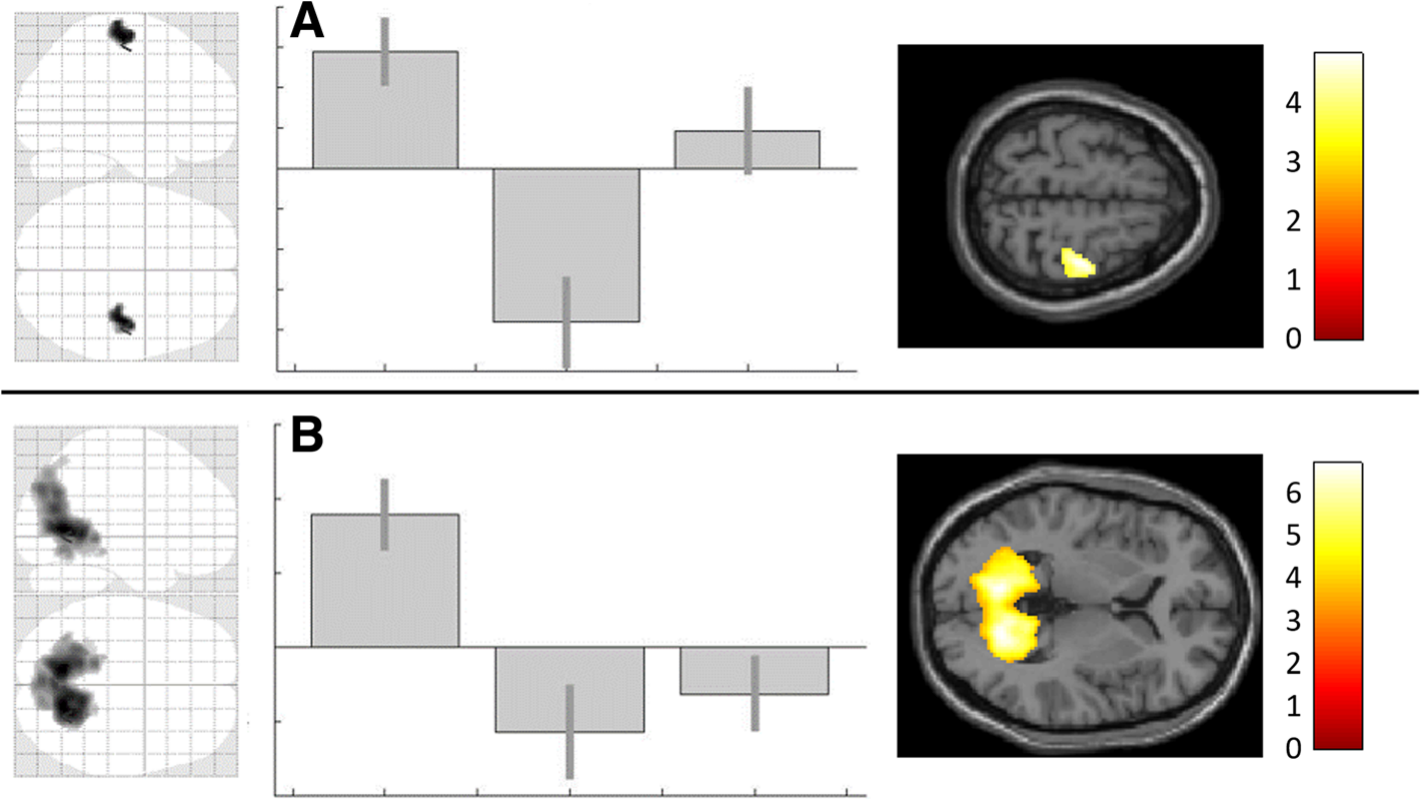
Polarity dependent effect of tDCS on visual and motor processing. Significant (p_FWE_ < .05) alterations of BOLD-response after DCS (postcathodal vs. postanodal) for A. motor- (precentral-gyrus), and B visual-processing (calcarine-gyrus). Illustrated as SPM generated glass-brain and T1-overlay; for better visualization both displayed with a threshold of p_unc_ < .0005. Corresponding contrast estimates in the following order: 1. postcathodal, 2. postanodal and 3. postsham. For coordinates and further details see Table 3

In both control conditions (visual- and motor-paradigm) the observed effect was antipodal to the effect on trigeminal nociception showing a decrease of the BOLD-response in the calcarine- (for visual stimulation) and right precentral-gyrus (for left hand motor activation) comparing post-anodal vs. post-cathodal activation (Table 3, Fig. 3). As in nociception no voxels showing the opposite modulatory patterns of activity could be identified.

**Table 3:** Effect of tDCS on motor and visual processing

MNI X Y Z	Anatomical area		k_E_	T
Visual				
20–64 4	BL	Calcarine gyrus	1848	6.62
-14–40 -4	L	Lingual gyrus	15	4.74
18–62 -10	R	Lingual gyrus	6	4.47
Motor				
40–18 62	R	M1/Precentral Gyrus	15	4.82

Areas with significant DC-stimulation induced alterations (postcathodal vs. postanodal) in both control paradigms. Illustration in Fig. 3. All results whole brain Family-Wise-Error corrected (p_FWE_ < 0.05). R = right, L = left, BL = bilateral, Ke = cluster extend, MNI = Montreal Neurological Institute

## Discussion

This study aimed to explore the underling mechanism of the previously described antinociceptive effects of tDCS targeting the primary motor cortex, and demonstrates polarity-specific effects of tDCS on specific brain regions associated with trigeminal pain processing.

Anodal tDCS increased BOLD-response in the thalamus, basal ganglia, cingulate cortex, dorsolateral prefrontal cortex and amygdala, whereas cathodal tDCS lead to a decrease of activation in these regions. These areas are involved in human trigeminal pain processing and were described to play a role in several pain disorders as well as experimental pain studies, with different contributions to pain perception and processing. Irrespectively of polarity behavioral data were not altered after tDCS. Cerebral activations for control paradigms (motor task and visual stimulation) were in the expected range and located within expected brain areas.

It is well-known that anodal tDCS leads to increased cortical excitability, while cathodal tDCS induces the opposite effect. Investigating the effects of tDCS Vaseghi et al. demonstrated that anodal tDCS over M1 enhances brain excitability for at least 30 min using motoric evoked potentials (MEP) [26]. Several other studies confirmed increased MEP after application of a-tDCS over M1 [4, 15, 27–29]. Our nociceptive data mesh with these results conclusively, showing increased activation BOLD signal after anodal stimulation.

More supporting evidence is coming from electrophysiological pain studies, showing that tDCS also modulates pain-related evoked potentials recorded after painful electrical stimulation of the forehead and the hand in healthy volunteers [30]. Cathodal tDCS generates inhibition of trigeminal and extracranial pain processing while a-tDCS leads to excitation. Similar results were observed investigating laser evoked potentials. After c-tDCS N2 amplitude and P2 components were significantly reduced compared with anodal and sham stimulation [31].

Only few data exist for functional imaging of pain processing after tDCS. Ihle et al. investigated 16 healthy volunteers in a randomized, cross-over sham controlled study using fMRI with an acute heat pain paradigm [32]. No significant polarity-specific changes of brain activation were observed comparing active with sham stimulation. When directly contrasting anodal and cathodal stimulation a decrease of activation in the hypothalamus, inferior parietal cortex, inferior parietal lobe, anterior insula, and precentral gyrus was observed in an uncorrected analysis. This changes interestingly were mainly observed on the contra-stimulus side (changes of activation mainly in the right hemisphere following right sided heat stimulation and left sided tDCS, p_unc_ < 0.001). Although anatomic structures similar to our study were affected, the effects showed opposite behaviour with a decrease of activation after anodal stimulation. It remains speculative, but as duration and site of stimulation was comparable in both studies, differences might be caused by differing current intensities and type of pain (heat stimulus vs. electrical stimulation). And indeed, there is evidence that different stimulation intensities may lead to antipodal cortical reaction regarding excitability [33].

As other experimental studies, we were not able to demonstrate a significant modulation of the recorded behavioral data. A meta-analysis including eight studies showed that c-tDCS of the primary motor-cortex leads to significant sensory threshold but not pain threshold alterations in healthy volunteers. Interestingly in chronic pain, pain-levels were significantly reduced [34]. No modulation of behavioral data caused by tDCS was detected by several other studies [31, 32] investigating acute pain. Taking this together TDCs may be able to modulate chronic but not acute and experimental pain.

Interpreting the results of motor processing our data are perfectly in line with the concept of intercortical/transcalosal inhibition [35] as tDCS was applied to the contralateral hemisphere (left M1) of the task based activation of the primary motor cortex (right M1, left hand), which was decreased after anodal stimulation.

The observed alterations in the visual system are difficult to interpreted and remain speculative. Recent evidence supports a complex relationship and influence between the here targeted nociceptive and the also affected visual central processing [36].

The underlying neurobiological mechanisms of tDCS are still unclear. Different mechanisms were previously discussed involving a cascade of events at cellular and molecular levels [37]. Local and distant neuroplastic changes were described [37]. Several neurotransmitters such as dopamine, acetylcholine, and serotonin are involved in this process [38, 39]. GABAergic neurotransmission via interneurons is modulated by tDCS [40]. Animal studies showed that anodal stimulation causes depolarization while cathodal stimulation causes hyperpolarization of neurons [41, 42] inducing an alteration of neural activity not only during tDCS but also hours later [41]. Pharmacological studies suggest that NMDA and GABAergic systems are involved in the underlying neurobiological mechanisms [40, 43, 44]. Additionally, spectroscopic data showed that cerebral GABA and glutamate concentrations were altered after tDCS application [45, 46].

Regarding the effect of tDCS on pain processing immediate after-effects and long-lasting effects have to be differentiated [47, 48]. TDCS-induced alterations of the acid-base balance of neuronal membranes were thought to play an important role for direct modulation of central pain processing leading to a reduction of NMDA receptor activity [49]. Long-lasting effects were thought to be mediated at a synaptic level by NMDA receptors in terms of long-term-potentiation (LTP) and depression (LTD) respectively [43, 44]. Further research suggested that also non-synaptic mechanisms might be involved in long-lasting effects of tDCS [47]. As a result of these molecular and cellular processes, modulation of functions of brain areas related to pain processing may occur. Previous animal experiments suggested that tDCS regulates neuronal activity by top-down modulation not only in the brain but also in the spinal cord. tDCS decreased brain-derived neurotrophic factor (BDNF) levels within the spinal cord and brainstem in areas involved in the descending pain processing system thereby decreasing pain sensitivity [50].

Hence, tDCS dependent pain reduction might be the result of combined modulation of the pain processing network and facilitation of descending pain inhibitory mechanisms. The widespread alterations in the central nociceptive processing identified in this study support this network based hypothesis. Until now it remains unclear whether the observed effects are different in patients suffering from pain as most of the studies investigated healthy volunteers. Animal data indicate that chronic stress might influence brain reaction to tDCS [50]. BDNF levels were only reduced by tDCS in unstressed animals. Therefore, the impact of DCS in patients might be significantly different from healthy subjects. Additionally, disorder-specific effects might be conceivable and contribute to the current heterogeneity of study results.

The here observed effects of tDCS may be of particular interest in the context of migraine, as anodal tDCS was previously shown to have alleviating effect on chronic and episodic courses of migraineurs [51–55]. At first glance this may be counterintuitive as migrainous brains, especially interictally, were proven to have a lack of habituation regarding multiple sensory modalities [56]. The current study demonstrates an even higher pain related activation immediate after single session a-tDCS. However treatment effects in clinical studies were only detected with delay and after several sessions. Furthermore, brains of migraineurs may react different from those of healthy controls, and migraine hyperresponsiveness is yet not fully understood as there is an ongoing debate, whether this is the result of a decreased inhibition, or a decreased pre-activation [56, 57]. To treat this hyperresponsiveness simply by means of inhibitory neuromodulation is probably too simply thought, as in fact treatment studies using DCS or TMS favor excitatory over inhibitory stimulation [51, 53–55, 58–61]. Demonstrating modulation not only localized cortical, but also subcortical and in remote structures hint towards a more complex and network wide modulation, which is further supported by our findings for motor activity and visual stimulation demonstrating tDCS’s reversed influence on even more remote networks.

A limitation of the study is that data were obtained from healthy young volunteers without any history of pain. Pain was artificially induced and not caused by a genuine pain disorder. Therefore, activation as well as modulation might be different in pain patients who may respond differently to tDCS. Additionally, only pain modulation after tDCS of M1 was investigated. Further research is needed regarding optimal tDCS application time, site of stimulation, current intensity and electrode size.

## Conclusion

We hereby demonstrate polarity-specific modulation of specific brain regions associated with cerebral pain processing using tDCS. Anodal tDCS led to an increase of activation within the cerebral pain-network while cathodal tDCS led to a decrease of activation. These findings support previous electrophysiological findings detecting an increase of cortical excitability after a-tDCS and a decrease after c-tDCS. The results enrich the understanding of the antinociceptive capabilities of tDCS as they point towards a network wide modulation of this system. Furthermore, the observations for motor activity and visual stimulation improve the knowledge regarding tDCS’s influence on even more remote networks, as for these the modulatory effect was reversed in the contralateral M1 and bilaterally in the visual cortices. Further studies need to evaluate whether these data can be transferred to patients with pain and headache disorders.

## Abbreviations

AAL: Automated anatomic labeling
ACC: Anterior cingulate cortex
a-tDCS: Anodal transcranial direct current stimulation
BDNF: Brain derived neurotrophic factor
BOLD: Blood oxygen level dependent
c-tDCS: Cathodal transcranial direct current stimulation
FWE: Family wise error correction
fMRT: Functional magnetic resonance imaging
LTD: Long term depression
LTP: Long term potentiation
M1: Primary motor cortex
MNI: Montreal neurological institute
MPRAGE: Magnetization prepared rapid acquisition gradient echo
NRS: Numeric rating scale
ROI: Region of interest
S1: Primary somatosensory cortex
S2: Secondary somatosensory cortex
SPM: Statistical parametric mapping
s-tDCS: Sham transcranial direct current stimulation
tDCS: Transcranial direct current stimulation
TMS: Transcranial magnetic stimulation
